# Evaluation of Potential Molecular Targets of the Alkaloid Epiisopiloturine, Involved in Cardioprotective Effects, Using Computational Molecular Docking in an Animal Model of Cardiac Ischemia and Reperfusion

**DOI:** 10.3390/ijms26199488

**Published:** 2025-09-28

**Authors:** Francisco Sandro Menezes-Rodrigues, Elisa Andrade Costa, Pedro Ivo De Marqui Moraes, Erisvaldo Amarante de Araújo, Carlos Eduardo Braga Filho, Leiz Maria Costa Véras, Paulo Sérgio de Araujo Sousa, Jefferson Almeida Rocha, Nelson Americo Hossne Junior, Solange Guizilini, Isadora S. Rocco, Walter José Gomes, Afonso Caricati-Neto, Marcelo Pires-Oliveira, Célia Maria Camelo Silva, Almir Gonçalves Wanderley, Fernando Sabia Tallo

**Affiliations:** 1 Postgraduate Program in Interdisciplinary Surgical Science, Universidade Federal de São Paulo (UNIFESP), São Paulo 04024-000, SP, Brazil; sandro.rodrigues@unifesp.br (F.S.M.-R.); andrade.elisa@unifesp.br (E.A.C.); fstallo@unifesp.br (F.S.T.); 2 Postgraduate Program in Cardiology, Universidade Federal de São Paulo (UNIFESP), São Paulo 04024-000, SP, Brazil; pedroivo_mm@hotmail.com (P.I.D.M.M.); biomedipatologia@icloud.com (E.A.d.A.); sguizilini@unifesp.br (S.G.); isadora.rocco@unifesp.br (I.S.R.); wjgomes1012@gmail.com (W.J.G.); 3 Department of Pharmaceutical Sciences, Universidade Federal de Pernambuco (UFPE), Recife 50740-520, PE, Brazil; 4 Research Center on Biodiversity and Biotechnology, Universidade Federal do Delta do Parnaíba (UFDPar), Parnaíba 64202-020, PI, Brazil; leiz@ufpi.edu.br (L.M.C.V.); psergio.araujosousa@gmail.com (P.S.d.A.S.); jeffersonbiotec@gmail.com (J.A.R.); 5 Medicinal Chemistry and Biotechnology Research Group (QUIMEBIO), Universidade Federal do Maranhão (UFMA), São Bernardo 65550-000, MA, Brazil; 6 Cardiovascular Surgery Discipline, Universidade Federal de São Paulo, São Paulo 04024-002, SP, Brazil; nelson.hossne@unifesp.br; 7 Department of Pharmacology, Universidade Federal de São Paulo (UNIFESP), São Paulo 04023-062, SP, Brazil; caricatineto@gmail.com; 8 Department of Medicine, Centro Universitário UNIME, Lauro de Freitas 42700-000, BA, Brazil; marpoliv@umich.edu; 9 Department of Pharmaceutical Sciences, Universidade Federal de São Paulo (UNIFESP), Diadema 09913-030, SP, Brazil

**Keywords:** cardiac diseases, cardiac ischemia–reperfusion, myocardial infarction, pharmacological cardioprotection, epiisopiloturine

## Abstract

The most common cause of morbidity and death worldwide is acute myocardial infarction (AMI), which is typified by severe and deadly arrhythmias resulting from cardiac ischemia and reperfusion (CIR). We chose to investigate the possible cardioprotective activity of epiisopiloturine (EPI), an imidazole alkaloid presents in the leaves of *Pilocarpus microphyllus*, in an animal model of CIR in rats. Control rats were treated with 0.9% saline solution and then subjected to CIR (SS + CIR); they were compared to rats pretreated with either 10 mg/kg (EPI10 + CIR group) or 15 mg/kg EPI (EPI15 + CIR) before CIR. ECG analysis was used to assess the incidence of ventricular arrhythmias (VAs), atrioventricular block (AVB), and lethality (LET) brought on by CIR in these rats. Serum creatine kinase-MB (CK-MB) was assessed using a colorimetric assay. In comparison to the SS + CIR group, animals treated with EPI15 + CIR had lower AVB incidence, which decreased from 85.7% to 21.4%, while LET incidence decreased from 71.4% to 21.4%. In both EPI10 + CIR and EPI15 + CIR groups, serum CK-MB was lower than in SS + CIR positive controls. These findings suggest that administration of EPI (15 mg/kg) before CIR could reduce the incidences of AVB and LET, as well as cardiac injury markers, which suggests that, likely due to its antioxidant effects, EPI may be a promising drug to reduce LET in patients with severe and fatal arrhythmia due to AMI.

## 1. Introduction

Cardiovascular diseases (CVDs) are a major cause of mortality in both developed and developing nations, contributing to almost 26 million deaths annually worldwide. Globally, the two most common causes of morbidity and death among these conditions are acute myocardial infarction (AMI) and coronary artery disease (CAD) [1,2,3,4]. AMI is one of the potentially lethal coronary-related conditions that is strongly associated with sudden cardiac death, which kills about three million people annually globally [3,5,6].

Oxygen deprivation in cardiac cells is the primary cause of permanent myocardial damage in AMI. This might affect diastolic and systolic function and increase the patient’s risk of developing severe and fatal cardiac arrhythmias [5,6,7]. Even though AMI can lead to several serious problems with heart function, there are currently few pharmaceutical alternatives for treating it. Treating AMI requires the quickest feasible reperfusion, or restoration of coronary blood flow after ischemia [5,6,7,8]. Early therapy (less than six hours after the beginning of symptoms) improves the outcome.

Treating AMI requires the quickest feasible reperfusion, or restoration of coronary blood flow after ischemia [5,6,7,8,9]. Early therapy (less than 6 h after the beginning of symptoms) improves the outcome. The main therapy for AMI is reperfusion of the myocardium that has been subjected to ischemia; nevertheless, this procedure can produce serious cardiac dysfunctions, mainly due to the cardiac ischemia and reperfusion (CIR) process’s rapid oxygen entry and severe ionic dysregulation in cardiac cells. Lethal ventricular arrhythmias that are directly linked to the disruption of intracellular Ca^2+^ homeostasis in cardiac cells can result from these dysfunctions [7,8,9,10,11,12,13].

*Pilocarpus microphyllus*, commonly known as jaborandi, is a species belonging to one of the most significant genera of the Brazilian flora with native representatives in the northeastern and northern regions of the country [14], and it has long been investigated due to its potential cardioprotective compounds [15]. Due to its substantial content of the imidazole alkaloid pilocarpine, which is widely used in the treatment of glaucoma, the regulation of xerostomia, and the stimulation of sweat and lacrimal glands, *P. microphyllus* Stapf (Rutaceae) stands out as the most economically important species within the Jaborandi group. Pilocarpine and other imidazole alkaloids, such as isopilosine, epiisopilosine, and epiisopiloturine (EPI), have been identified in *P. microphyllus* [16,17,18,19]. Among its documented biological activities, the antiparasitic, anti-inflammatory, and antioxidant properties of EPI stand out, having been widely reported in the literature and associated with diverse pharmacological effects [20,21,22,23].

The aim of this study was to evaluate the potential cardioprotective and antiarrhythmic effects of EPI, given the scarcity of existing data on its pharmacological activity in the cardiovascular system. To this end, a classical animal model of cardiac ischemia–reperfusion (CIR) was used to investigate the impact of EPI treatment on the incidence of arrhythmias, specifically ventricular arrhythmias (VAs) and atrioventricular block (AVB), as well as on lethality (LET), serum levels of the cardiac injury biomarker CK-MB, and histological evidence of myocardial damage in rats subjected to CIR.

## 2. Results

### 2.1. Computational Studies

**Figure 1:**
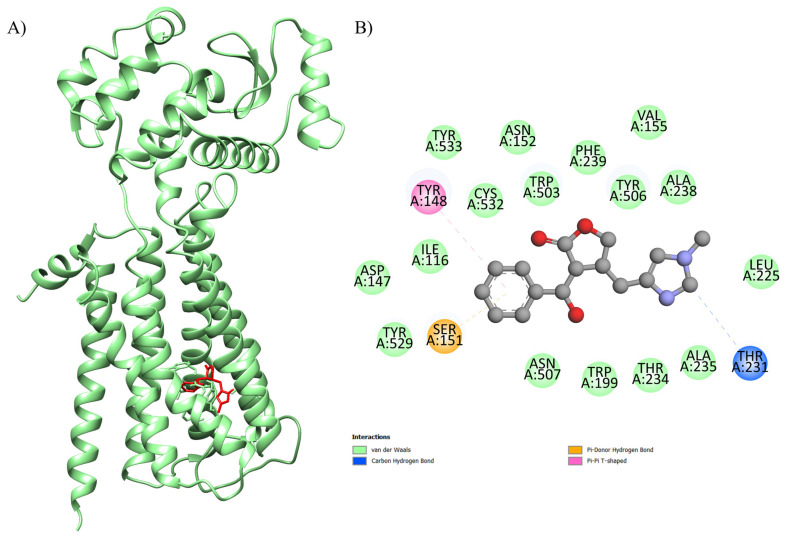
Three-dimensional molecular docking of the ligand–protein complex with 4U14 (color: green) and EPI (color: red) illustrating the (**A**) active binding site with the (**B**) respective interactions.

The results regarding the molecular docking of EPI with the proteins are presented in Table 1. The optimal binding energy parameters were obtained from the interaction between the EPI and 4U14 (code 4U14 corresponds to the type 3 muscarinic receptor—M3), EPI and 5ZK8 (code 5ZK8 corresponds to the type 2 muscarinic receptor—M2), EPI and 6DT0 (code 6DT0 corresponds to the mitochondrial calcium uniporter—MCU), EPI and 8E59 (code 8E59 corresponds to the L-type Ca^2+^ channel—LTCC), and EPI and 7KL5 (code 7KL5 corresponds to the type 2 ryanodine receptor—RYR2). The affinity was observed with a binding energy equal to −8.6 kcal.mol^−1^, −8.4 kcal.mol^−1^, −7.6 kcal.mol^−1^, −8.6 kcal.mol^−1^, −6.8 kcal.mol^−1^, and −5.6 kcal.mol^−1^, respectively (Table 1; Figure 1). In the formation of this complex, it was possible to observe interactions with the amino acids Ser A: 151, Tyr A: 529, Asp A: 147, Ile A: 116, Tyr A: 148, Tyr A: 533, Cys A: 532, Asn A: 152, Trp A: 503, Phe A: 239, Tyr A: 506, Val A: 155, Ala A: 238, Leu A: 225, Thr A: 231, Ala A: 235, Thr A: 234, Trp A: 199, and Asn A: 507 from the active site of the protein with EPI.

### 2.2. Incidence of VA, AVB and LET Induced by CIR

**Figure 2:**
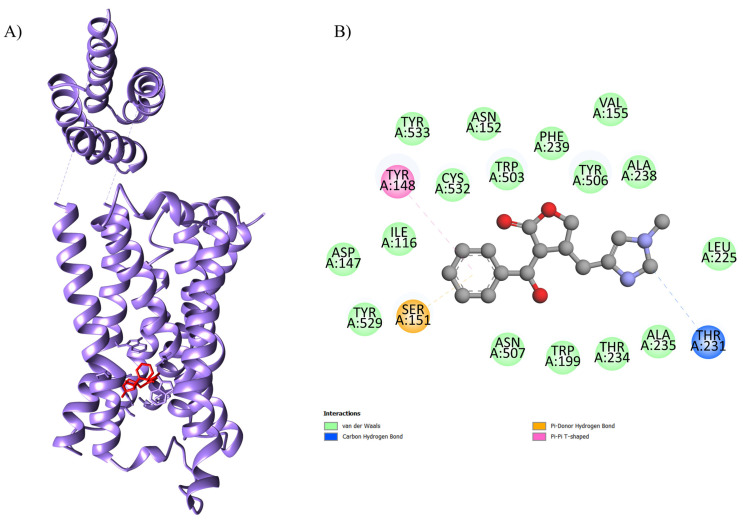
Three-dimensional molecular docking of the ligand–protein complex with 5ZK8 (color: purple) and EPI (color: red) illustrating the (**A**) active binding site with the (**B**) respective interactions.

**Figure 3:**
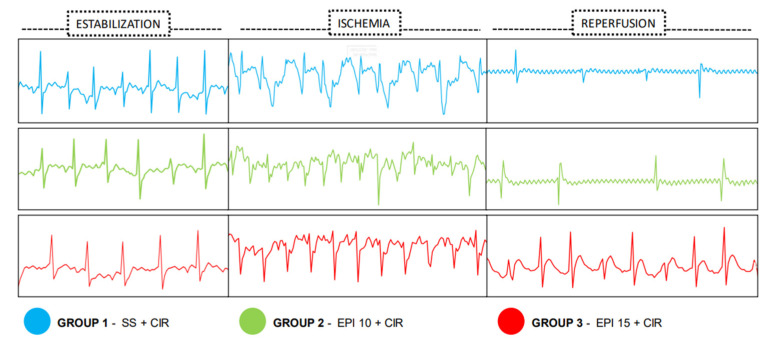
(Blue) rats treated with saline solution (SS) as controls had their ECG recorded before the cardiac ischemia and reperfusion (CIR) protocol. (Green) rats treated with 10 mg/kg EPI (EPI10 + CIR) before the CIR protocol. (Red) rats treated with 15 mg/kg EPI (EPI15 + CIR) before the CIR protocol.

**Figure 4:**
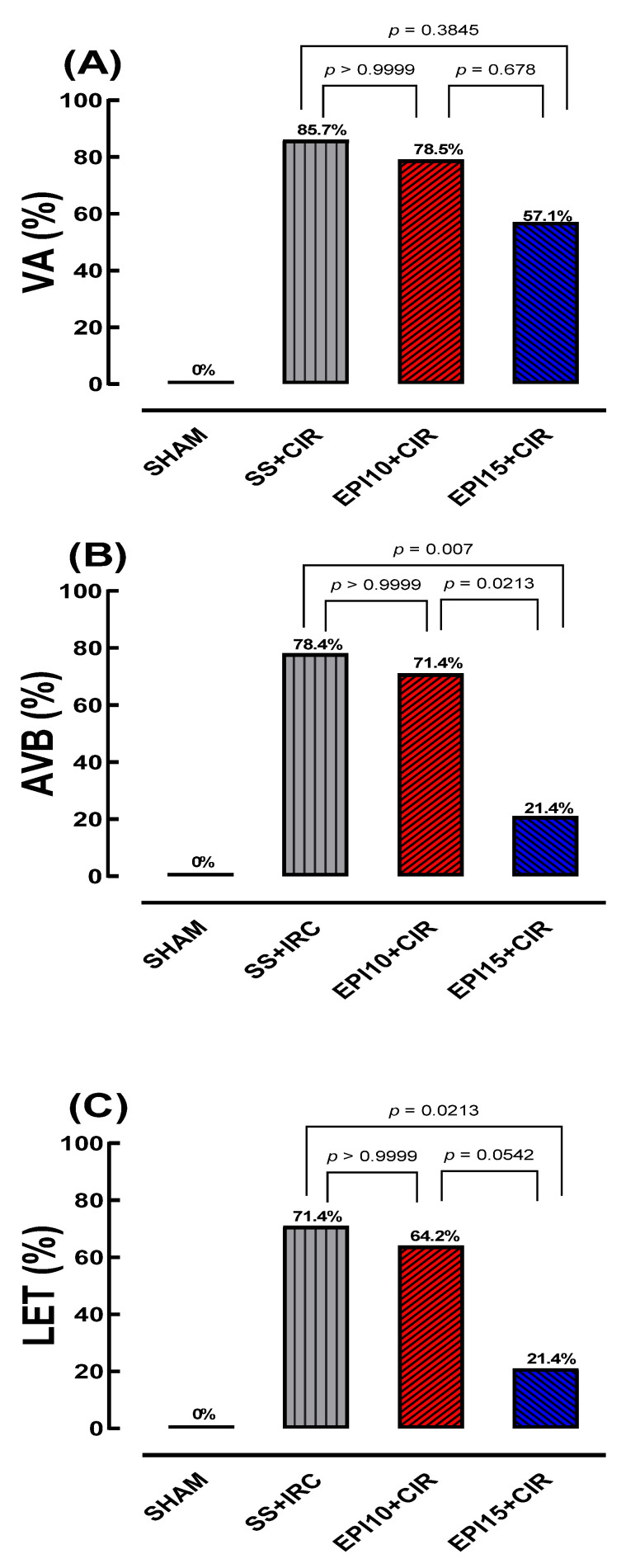
Histograms represent the (**A**) incidence of ventricular arrhythmias (VAs), (**B**) atrioventricular block (AVB), and (**C**) lethality (LET) in the SHAM, CIR, EPI10 + CIR (10 mg/kg EPI), and EPI15 + CIR (15 mg/kg EPI) groups. Results were expressed as percentage incidences, and statistical analysis was performed using Fisher’s exact test. The incidences of AVB and LET were significantly reduced in animals treated with EPI15 + CIR, but not EPI10 + CIR, when compared to SS-CIR. SHAM = (sham-operated) group; SS + CIR group: NWRs were pretreated with 0.9% saline solution (SS) intravenously and then subjected to cardiac ischemia and reperfusion (CIR); EPI10 + CIR group: NWR were pretreated with i.v. 10 mg/kg EPI prior to CIR; EPI15 + CIR group: NWRs were pretreated with i.v. 15 mg/kg EPI prior to CIR.

Before CIR protocol (stabilization periods for 15 min), no cardiac arrhythmias were detected in all animals studied. But, during CIR protocol, VA and AVB incidence in different experimental groups were detected through ECG and measured (Figure 2). Figure 3 shows that the VA, AVB, and LET incidence in the EPI10 + CIR group was not different to the SS + CIR group, indicating that the pretreatment with EPI 10 mg/kg (IV) did not reduce the VA, AVB, and LET incidence induced by SS + CIR. However, these incidences were significantly reduced in the EPI15 + CIR group compared to the SS + CIR group, indicating that pretreatment with EPI 15 mg/kg (IV) was effective in reducing cardiac arrhythmia (AVB) and LET. In the EPI15 + CIR group, the AVB and LET incidences were reduced from 70% to 20%, compared to the SS + CIR group (See Figure 3 and Figure 4).

### 2.3. Effects of the EPI on CK-MB Levels in Rats Under CIR

**Figure 5:**
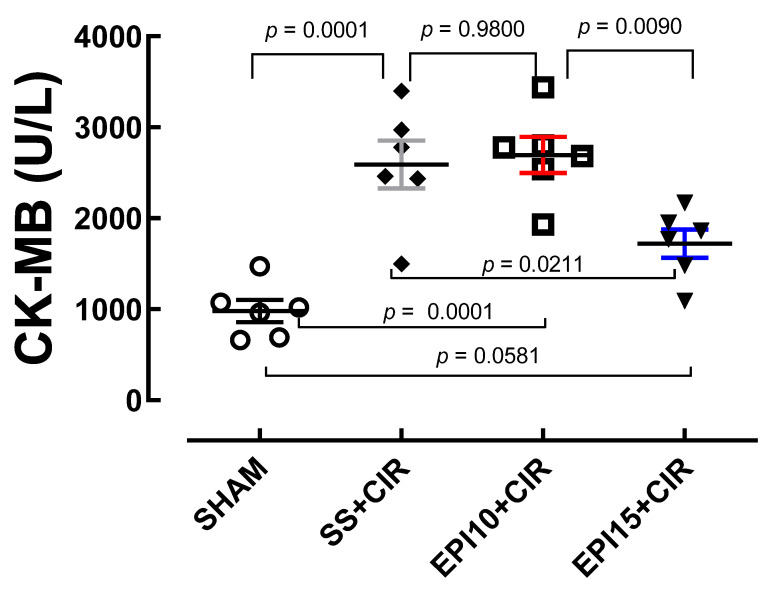
Serum concentration of CK-MB (U/L) of the SHAM, SS + CIR, EPI10 + CIR, and EPI15 + CIR groups. The results were expressed as mean ± standard error of the mean (*n* = 6) and analysis of variance (ANOVA) was applied, followed by Tukey’s post-test. SHAM (sham-operated) group; SS + CIR = group treated with saline and subjected to cardiac ischemia and reperfusion; EPI10 + CIR = group treated with 10 mg/kg EPI and then subjected to cardiac ischemia and reperfusion; EPI15 + CIR = group treated with 15 mg/kg EPI and then subjected to cardiac ischemia and reperfusion. SS + CIR and EPI10 + CIR rats had higher CK-MB than sham controls (*p* < 0.0001); EPI15 + CIR treatment significantly reduced CK-MB when compared to SS + CIR rats (*p* = 0.0211), resulting in levels like SHAM controls (*p* = 0.0581).

In Figure 5, we can observe that the serum concentrations of CK-MB of the SS + CIR (2591 ± 262.7 U/L, *n* = 6) and EPI10 + CIR (2695 ± 198.4 U/L, *n* = 6) groups were statistically higher than that of the EPI15 + CIR group (1720 ± 156.2 U/L, *n* = 6); moreover, all other groups presented serum concentrations of CK-MB higher than the SHAM group (979.7 ± 121.3 U/L, *n* = 6).

### 2.4. Myocardial Histopathological Analysis

When compared to the SS + CIR group, Figure 4 illustrates that the myocardial tissue exhibited opposing histopathological effects from the treatment of EPI10 and EPI15 prior to cardiac ischemia and reperfusion. The cardiac tissue in the SHAM group has striated cells with well-centralized nuclei that are normal in size and color, and there is no necrosis present. The cardiac tissue in the SS + CIR group exhibits severe coagulation-induced necrosis, with pyknotic cells and a significant number of cells undergoing karyolysis, swelling, and vacuolization. Similar results are seen in the EPI10 + CIR group, including coagulation-induced necrosis, severe tissue loss, myocytolysis-affected regions, swelling, vacuolated muscle fibers, and cells with decreased nuclei or in karyolysis. The EPI15 + CIR group, on the other hand, has weaker coagulation-induced necrosis, with minimal pyknosis, mild karyolysis, swelling, and no vacuolization, although a significant portion of the striated cells are preserved.

## 3. Discussion

The data obtained from this study demonstrate that pretreatment of animals submitted to CIR with EPI 15 mg/kg, intravenously, reduced the AVB incidence due to an important antiarrhythmic effect and then reduced the LET incidence (see Figure 3 and Figure 4). This is, to our knowledge, the first experimental study to show evidence of the cardioprotective effects of EPI, an imidazole alkaloid isolated from *P. microphyllus.*

Regarding serum CK-MB levels measured in the different groups studied, our results demonstrate that the CIR protocol used in this study caused a statistically significant increase when compared to the serum concentration of CK-MB when compared to the SHAM group. The same occurred with EPI + CIR when compared to the SHAM group. Thus, we can observe that the treatment of NWRs with EPI 15 mg/kg was able to reverse the increase in serum CK-MB values observed in the SS + CIR and EPI + CIR groups (Figure 5).

Table 1 displays our findings for the molecular docking of EPI with the targets studied in our hypotheses. The interaction between the EPI and 4U14 (PDB code: type 3 muscarinic receptor, M3), EPI and 5ZK8 (PDB code: type 2 muscarinic receptor, M2), EPI and 6DT0 (PDB code: mitochondrial calcium uniporter, MCU), EPI and 8E59 (PDB code: L-type Ca^2+^ channel, LTCC), and EPI and 7KL5 (PDB code: type 2 ryanodine receptor, RYR2) yielded noteworthy binding energy values (Gibbs free energy, ΔG) in our in silico approach. These were binding energies of −8.6 kcal.mol^−1^, −8.4 kcal.mol^−1^, −7.6 kcal.mol^−1^, −8.6 kcal.mol^−1^, −6.8 kcal.mol^−1^, and −5.6 kcal.mol^−1^, respectively (Table 1 and Figure 1).

**Figure 6:**
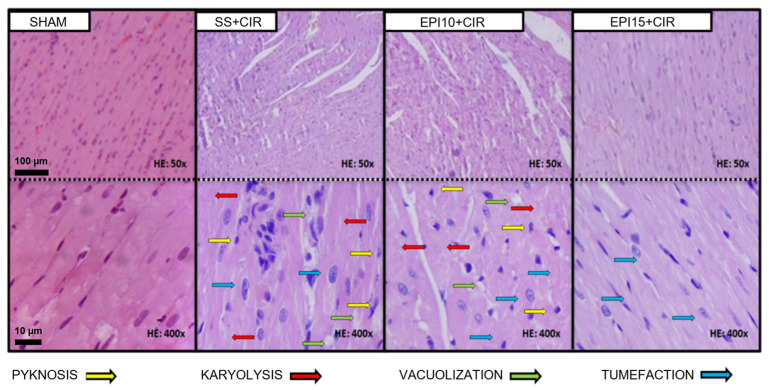
Examination of the myocardial histopathologically in animals from several experimental groups. Histopathological studies of the rat heart from the SHAM, SS + CIR, EPI10 + CIR, and EPI15 + CIR groups are shown in representative photomicrographs. The myocardium in the SHAM group is distinguished by striated cells with well-centralized nuclei of normal size and color, as well as the lack of necrosis. The SS + CIR group has severe coagulation-induced necrosis, characterized by pyknotic cells (decentralized nuclei and chromatin condensation), a significant proportion of cells experiencing karyolysis (nucleus absence and cytoplasmic eosinophilia), swelling, and vacuolization. Like this, the EPI10 + CIR group exhibits severe coagulation-induced necrosis, severe tissue loss, myocytolysis-affected regions, and swollen muscle fibers (an increase in cell volume brought on by the buildup of water (reversible owing to ionic imbalance)), vacuolation, and cells that are karyotic or have smaller nuclei. Nevertheless, the myocardium in the EPI15 + CIR group exhibits modest coagulation-induced necrosis, along with mild karyolysis, swelling, lack of vacuolization, and preservation of a significant portion of the striated cells (HE at 400× magnification).

Some studies have suggested that EPI is able to reduce the amount of Ca^2+^ in the cytoplasm of neutrophils in a sustained manner, and then significantly reduce the cellular collapse generated by the increment of intracellular Ca^2+^ concentration. In fact, compounds that have the imidazole ring in their chemical structure, such as antifungal drugs belonging to the azoles class itraconazole, miconazole and ketoconazole, can inhibit the Ca^2+^ influx through blocking plasma membrane Ca^2+^ channels and can inhibit the displacement of intracellular Ca^2+^ into the cytosol of mammalian cells [20,21,22,23]. These cellular actions of EPI may have cardioprotective benefits in the cardiovascular system because they pharmacologically modulate the Ca^2+^/cAMP/ADO signaling in cardiac cells. Our research group’s studies and other pertinent studies [7,8,13] have shown that a common and successful pharmacological strategy used to eliminate cardiac arrhythmias in a variety of clinical settings, particularly cardiac surgery, is the attenuation of cytosolic Ca^2+^ overload produced by the blocking of Ca^2+^ influx mediated by the L-type Ca^2+^ channel (LTCC) and/or blocking the ryanodine receptor 2 (RYR 2), which is located in the membrane of the sarcoplasmic reticulum of the cardiomyocyte, and stimulation of cardiac A1-adenosine receptors (A1R) produced by endogenous adenosine (ADO) and other agonists of A1R [8,13,24]. Because the cytosolic Ca^2+^ excess is attenuated, there is a decreased chance of excitation–contraction coupling (CECC) mismatch, which lowers the frequency of arrhythmias. These EPI effects may avoid contractile dysfunctions of cardiac cells (Figure 6).

Apart from its function in CECC, cAMP production by AC isoforms 5 (AC5) and 6 (AC6) is modulated by LTCC-mediated Ca^2+^ influx in cardiac cells. Furthermore, intracellular cAMP production and efflux are increased by pharmacological blocking of Ca^2+^ via LTCC [8,9,10]. The paradigm for Ca^2+^-mediated inhibition of AC5 and AC6 in the submicromolar range has been established through biochemical investigations of membrane preparations in overexpression systems, isolated organs, and in vivo experiments [12,13,25,26,27].

ADO, which is produced in the extracellular media from cAMP, can activate A1R in the cardiac cell’s plasma membrane to precisely control cardiac activity [12,13]. In a variety of therapeutic settings, including cardiac surgery, A1R stimulation combined with ADO is a frequently employed and successful tactic for eliminating cardiac arrhythmias [12,13,24,25,26,28,29,30,31]. According to our proposal, it may be possible to prevent sudden death in AMI patients by pharmacologically modulating the Ca^2+^/cAMP/ADO signaling in cardiac cells. We believe that EPI may promote a multitarget action by blocking both LTCC and RYE2, which results in a decrease in sarcoplasmic calcium, disinhibition of adenylate cyclase isoforms, and increased production of the second messenger cAMP, triggering an increase in the efflux of this second messenger and an increase in the amount of adenosine to cardiomyocytes, which increases the antiarrhythmic effect exerted by adenosine through the activation of A1R [32,33,34,35].

Furthermore, recent published studies also suggest that EPI can block the phosphorylation of the p65 subunit of NF-κB and, therefore, inhibits its translocation to the cell nucleus, where this dimeric protein composed of two subunits (p50 and p65) acts by promoting modulation of gene expression and protein synthesis and, consequently, its action on gene regulation [22]. Most likely, this inhibitory effect on NF-κB is of great importance considering that it is a fundamental step regarding the role that NF-κB plays in the inflammatory process, as it is from this process that this molecule regulates the genes responsible for the expression of pro-inflammatory cytokines tumor necrosis factor-alpha and interleukins (TNF-α, IL-1β, and IL-6), adhesion molecules, chemokines, growth factors, cyclooxygenase-2, and inducible nitric oxide synthase [22,23,36]. In addition, pretreatment with EPI significantly reduced the levels of nitrite produced by cultured microglial cells treated with LPS, which demonstrates the anti-inflammatory effect of EPI, which, at least in part, is related to the ability of EPI to promote inhibition of intracellular pathways responsible for the pathophysiology of inflammation, such as NF-κB and MAPKs. Furthermore, EPI was also able to inhibit the synthesis of the enzyme inducible nitric oxide synthase (iNOS), significantly reducing the production of inflammatory cytokines such as interleukin-1β (IL-1β), (IL-6), and tumor necrosis factor-alpha TNF-α when compared to the group of microglial cells treated with lipopolysaccharide (LPS) [22,23].

There is a correlation between increases in reactive oxygen species and inflammation marked by increased levels of malondialdehyde (MDA), TNF-α, and toll-like receptors in patients with surgery-induced cardiac damage [37]. Reactive oxygen species are similarly increased in myocardial infarction patients [38], in vivo or in vitro models of amiodarone cardiotoxicity [39], and other cardiovascular diseases [40]. These findings support the theory that the reduction in AVB and LET in rats treated with EPI and then put through the CIR regimen may be caused by the antioxidant and anti-inflammatory properties of EPI.

The current study bolsters the hypothesis that EPI, an imidazole alkaloid isolated from *P. microphyllus*, may exert a cardioprotective effect through its action on Ca^2+^/cAMP/ADO signaling in cardiac cells. Additionally, EPI may exert a cardioprotective effect by promoting the production of the anti-inflammatory cytokine IL-10 and decreasing the production of the pro-inflammatory cytokine IL-1β, making it a promising agent for the treatment of acute myocardial infarction.

As previously described, our data obtained from molecular docking also demonstrated that muscarinic receptors (M2 and M3) are potential molecular targets of EPI, in addition to the molecular targets already mentioned and described previously, which is why we believe that EPI promotes its diverse and different effects through multiple mechanisms of action. A study on the chronotropic and inotropic effects of three antagonists and four agonists of mAChRs in isolated guinea pig atria was published by Chassaing et al. [41]. They postulated that there are two functional cardiac mAChR subtypes: one mediating the regulation of heart rate and the other mediating the contraction of the heart. These two groups were based on the observed variations in the potencies and efficacies of these substances in terms of their effects on heart rate and contractile force. But Clague et al. [42] re-evaluated the effects of agonists and antagonists on guinea pigs’ atrial rate and contraction in comparison to their ileal contractions. It was discovered that the variations in agonist potencies between these two tissues might be attributed to either variations in intrinsic efficacy or variations in sensitivity to acetylcholinesterase activity. It was determined that the slight variations in agonist potency between ileal and atrial muscarinic receptors were insufficient to support the idea of receptor heterogeneity. The work published by Jaiswal et al. [43] offers the first proof that mammalian hearts contain functional M3 receptors.

The researchers showed that a low dosage of 4-diphenylacetoxy-N-methylpiperidine methiodide (4-DAMP, 10 nM) blocked the effect of ACh’s enhanced prostaglandin synthesis in the isolated rabbit heart. Even though the researchers thought of 4-DAMP as an M2 antagonist, the dose at which it was utilized would probably block M3 receptors while having no effect on M2 receptors. After reassessing prostacyclin synthesis in rabbit hearts, the authors have concluded that ACh can act through the M3 receptors in ventricular myocytes. They discovered that hexahydro-sila-difenidol hydrochloride (HHSiD) and methoctramine (AF-DX 116), but not pirenzepine, decreased the synthesis of 6-keto-postaglandin (1 alpha) in ventricular myocytes that were stimulated by Ach [44].

Furthermore, AF-DX 116 was the only medication to reduce the ACh-induced decrease in isoproterenol-stimulated cAMP buildup; HHSiD and pirenzepine did not have this effect. Pertussis toxin (PTX) prevented the reduction in cyclic AMP caused by ACh (which is in line with the M2 receptor-Gi protein coupling), but it had no effect on the prostaglandin synthesis caused by ACh (which is in line with the Gq protein coupling). These findings provide compelling evidence that functional M2 and M3 receptors coexist in rabbit ventricles. Increases in IP formation in rat and guinea pig cardiomyocytes have been well-established by multiple groups [45,46,47]. This is a typical response to stimulation of the M1, M3, or M5 receptors, but not of the M2 or M4 receptors.

The mAChR-mediated PI hydrolysis in the ventricles and atria of guinea pigs was examined by Ford et al. [45] An affinity profile skewed from the pure M2 responses was produced by the effects of various antagonists, such as HHSiD and hexahydro-sila-difenidol hydrochloride (p-F-HHSiD), indicating “a second population of muscarinic sites” [45]. In neonatal rat ventricular myocytes, Sun et al. [46] investigated the antagonistic relationship between IP accumulation and carbachol-induced chronotropy. They discovered that AF-DX 116 and pirenzepine had no effects, but HHSiD prevented the effects of carbachol. They concluded that the population of muscarinic receptors in neonatal ventricular myocytes is diverse and includes both M2 and M3 subtypes. More proof that adult rat ventricular myocytes have functional M3 receptors was recently shown by Pönicke et al. [47]. The authors evaluated IP production in isolated myocytes produced by carbachol.

They discovered that pretreatment with PTX greatly increased the IP production generated by carbachol. This effect was countered by darifenacin, an M3-selective inhibitor [48] with a pKi value of 8.7, but was unaffected by himbacine, pirenzepine, or AF-DX 11. According to the author’s findings, the M3 subtype is present in adult rat cardiomyocytes and is linked to phospholipase C/inositol triphosphate (PLC/IP3) pathway activation. The presence of M3 receptors in the mouse atrial is further supported by functional data. In isolated mouse atria, researchers discovered a biphasic response to ACh that could both be blocked by atropine. The initial response was a temporary negative inotropic effect, which was followed by a positive inotropic impact [49,50].

The results of a thorough analysis supported the theory that an M3 subtype in mouse atria mediates positive inotropic effects induced by mAChR agonists. The negative inotropic response was found to be sensitive to PTX and could be countered by the M2-selective antagonist gallamine, while the positive inotropic response was inhibited by the M3-selective antagonist p-FHHSiD [49,50].

Therefore, based on all the data obtained and effects demonstrated through our study, we believe that EPI has considerable potential to become an important pharmacological tool in the future with valuable effects related to the management of heart disease patients, such as being antiarrhythmic, antioxidant, and cardioprotective.

## 4. Materials and Methods

### 4.1. Computational Studies

#### 4.1.1. Drawing and Optimization of EPI

The three-dimensional (3D) structure of EPI was designed and optimized using GaussView 5 and Gaussian 09w software, respectively. The optimization was carried out employing the Density Functional Theory (DFT) method with the hybrid B3LYP functional and the 6-311++G (d,p) basis set [20].

#### 4.1.2. Molecular Docking

The 3D structures of proteins were obtained from the Protein Data Bank (PDB) with the codes 5ZK8 (corresponds to the type 2 muscarinic receptor—M2), 4U14 (corresponds to the type 3 muscarinic receptor—M3), 6DT0 (corresponds to the mitochondrial calcium uniporter—MCU), 7KL5 (corresponds to the type 2 ryanodine receptor—RYR2), and 8E59 (corresponds to the L-type Ca^2+^ channel—LTCC). All preparation procedures for the ligand and macromolecules were conducted using AutoDockTools version 1.5.6. Gasteiger partial charges were calculated after adding polar and non-polar hydrogen atoms to the macromolecules and ligand. A cubic box of 30 × 30 × 30 Å was generated at the protein active site to define the ligand–protein interaction region. For each computational simulation using AutoDock Vina, the maximum number of binding poses, defined as “num_modes,” was set to 100, and the computational effort parameter, known as “exhaustiveness,” was set to 50. Interaction results were expressed as Gibbs free energy (ΔG), and clusters of complexes with the lowest interaction energies were selected for more detailed analysis of alkaloid–macromolecules interactions using BIOVIA Discovery Studio 2021 Client version v21.1.0.20298 and Chimera 1.14.

### 4.2. Animals

Normal male Wistar rats (NWRs), weighing between 280 and 320 g, were housed at 21 ± 2 °C with a 12:12 h light/dark cycle and provided with unlimited food and water. The Ethics Committee of the Escola Paulista de Medicina—Universidade Federal de São Paulo authorized all experimental protocols employed in this investigation (UNIFESP #9447210317 of 21 August 2017 and #7323080822 of 1 September 2022).

### 4.3. Cardiac Ischemia and Reperfusion (CIR) Induction

To reproduce in the laboratory an animal model of AMI, rats were submitted to surgical procedures in accordance with the methodology previously reported by our research group [8,13]. Rats were first anesthetized with intraperitoneal 100 mg/kg ketamine, 10 mg/kg xylazine, and 2 mg/kg tramadol. Then, rats were fixed in the supine position on a surgical platform heated by a thermal blanket, and the temperature was routinely monitored using a rectal thermometer to maintain it at 37.5 ° C. The animals were maintained under mechanical ventilation with a volume of 12 mL/kg and a rate of 70/min using a respiratory pump (model: Insight EFF 312, Insight Equipamentos Cientificos, Ribeirao Preto, Brazil). First, venous access was performed through the femoral vein with placement of a catheter to administer the drug at the appropriate time. Subsequently, orotracheal intubation was performed and the animals were subjected to mechanical ventilation with room air with a tidal volume of approximately 6 mL/kg of body weight and a respiratory frequency of 90 cycles per minute.

After the trichotomy, a left thoracotomy was performed between the 4th and 5th intercostal spaces. After breaking the pericardium, the heart was exteriorized by lateral compression of the chest, and under the left descending coronary artery a 4-0 suture ((4/0 braided silk suture coupled to a 10 mm micropoint reverse cutting needle, Ethicon K-890H, Cincinnati, OH, USA) was passed approximately 2 mm from the origin between the edge of the left atrium and the sulcus of the pulmonary artery [8,13]. Next, the heart was quickly placed back into the thoracic cavity, and the chest was closed. To perform the coronary ligation, the two ends of the nylon thread were passed through a cylindrical polypropylene tube, which was used to perform ischemia. After 15 min of stabilization, the tube was pressed over the coronary artery, and the nylon thread was pulled, and the set (nylon thread and tube) was fixed with Kelly forceps. To perform reperfusion, it is only necessary to detach this structure and remove the tube and nylon thread. After 10 min of myocardial ischemia, the tourniquet was removed to enable 75 min of coronary [8,13].

For the SHAM group the procedures followed as previously described; however, the nylon thread was only passed under the left coronary artery, and no coronary ligation was performed, thus neither ischemia nor reperfusion were induced. After surgery, ECG monitoring was maintained throughout the experimental period. Different experimental protocols were adopted as described below.

### 4.4. Assessment of Cardiac Activity During CIR

Electrocardiogram (ECG) analysis was used to assess the cardiac activity during CIR, using a protocol that our research group has previously published [8,13]. The cardioprotective effect of calcium channel blockers and other medications on the incidence of cardiac arrhythmias (VA and AVB) and lethality (LET) due to CIR was assessed in additional studies using this high-resolution methodology [8,13]. The ECG was initially recorded for 15 min before the stabilization phase and for 10 min during the 75 min-long ischemia and reperfusion protocols [8,12,13]. A biopotential amplifier and needle electrodes that were subcutaneously inserted into the limbs were used to record the ECG. The effective coronary artery was confirmed by using changes in the ECG (increase in the R wave and ST segment) brought on by CIR.

Using a heated operating table and the proper heating lamps, the body temperature was kept at 37.5 °C, and a rectal thermometer was used often to check the temperature. A computer system consisting of AqDados 7.02 hardware (Lynx Tecnologia Ltd., São Paulo, Brazil) and AqDAnalysis 7 software was used to process ECG data [8,13]. We were able to monitor heart rates using this technique in addition to the incidence of CIR-induced VA, AVB, and LET. Torsades, atrial fibrillation, and ventricular fibrillation were all classified as VA [12,13].

### 4.5. Biochemical Assessment of Heart Lesions’ Biomarkers

Using the approach outlined in our earlier investigations [11], the serum levels of the heart damage biomarkers CK-MB were measured. The blood samples came from rats who survived the entire 75 min CIR therapy. These 4–5 mL samples, which were taken from the abdominal aorta and stored in siliconized tubes, were centrifuged for 40 min at 5 °C at 2500 rpm. For the enzymatic measurement of CK-MB at 340 nm, the supernatant was extracted and kept at −20 °C. A kinetic UV test kit (Vida Biotecnologia Inc., Belo Horizonte, Brazil), was used for this purpose.

### 4.6. Histopathological Analysis of Left Ventricle Myocardial Tissue

Following the CIR surgical procedure, the abdominal cavity was opened, and arterial blood was drawn by accessing and puncturing the abdominal aorta. Following blood collection, an incision was made all the way down the sternum to access the thoracic cavity. After the circulatory vessels were severed from the heart using sharp scissors, the heart was removed completely. It was then immediately cleaned with saline solution, put in a vial containing 20 mL of buffered 10% formalin, and sent to the Histocell pathology laboratory (Sao Paulo, Brazil) for analysis.

A cross-section at the height of the lower atrial border was then used to separate the ventricles and atria, and the portion that included the atria and other nearby structures was thrown away. The left ventricle fragment was once more divided into three portions (the apex, mid, and distal regions) of roughly comparable thickness using axial cross-sections. These sections were then dehydrated for one hour each in increasing ethanol concentrations. Following two one-hour xylol washes to diaphanize the samples, they were twice washed for one hour and then blocked in paraffin at 60 °C. After cutting the material into cross-sections that were 4–5 µm thick, they were fixed with Mayer’s albumin and cleaned in water at 45 °C. Tissue slices were deparaffinized and stained with hematoxylin-eosin (HE) following two hours of heating at 60 °C.

A qualified pathologist who was blind to the different groups used an optical microscope (×400 and ×1000) to photograph the midsection of the left ventricle myocardium in the hearts of the animals in each group for descriptive analysis. Lesions associated with CIR were assessed to include the presence of hyperemic blood vessels, pyknosis, inflammatory infiltration, cardiomyocyte degeneration, loss of striation, and interstitial edema.

### 4.7. Pharmacological Treatments

EPI used to carry out this study was obtained from the residue obtained from the extraction of pilocarpine from the leaves of *P. microphyllus* [19]. The organic phase was subjected to liquid–liquid extraction, alkalinized with ammonium hydroxide solution to precipitate EPI in neutral form, and then the solution was filtered under reduced pressure. Then, to remove all impurities, EPI was subjected to the purification process by high-performance liquid chromatography (HPLC). Analyses were performed on a Shimadzu Prominence system equipped with a UV–Vis SPD-20A detector and a column oven CTO-20A set at 50 °C. A Lichrospher 60 RP-Select B column (5 µm) was used with an isocratic mobile phase of 5% potassium phosphate buffer at a flow rate of 1.0 mL/min, injection volume of 400 µL, and detection wavelength of 216 nm, proving EPI to be pure by HPLC (>95%) and showing data consistent with literature values [19]. To reduce the incidence of VA, AVB, and LET brought on by CIR, we administered EPI intravenously (IV) at doses of 10 mg/kg and 15 mg/kg to rats prior to CIR. The animals employed in this study were split up into the following experimental groups:

(1) SHAM (sham-operated) group (*n* = 10): Rats were subjected to all CIR procedures, except for myocardial reperfusion and the use of a left descending coronary artery tourniquet. They were also subjected to ECG analysis to ascertain the incidence of VA, AVB, and LET.
(2) SS + CIR group (*n* = 14): NWRs were pretreated with saline solution (SS) 0.9% intravenously and then submitted to cardiac ischemia and reperfusion (CIR). They were also subjected to ECG analysis to ascertain the incidence of VA, AVB, and LET.
(3) EPI10 + CIR group (*n* = 14): NWRs were pretreated with 10 mg/kg EPI intravenously prior to cardiac ischemia and reperfusion (CIR). They were also subjected to ECG analysis to ascertain the incidence of VA, AVB, and LET.
(4) EPI15 + CIR group (*n* = 14): NWRs were pretreated with 15 mg/kg EPI intravenously prior to cardiac ischemia and reperfusion (CIR). They were also subjected to ECG analysis to ascertain the incidence of VA, AVB, and LET.

In all experimental groups, LET was the primary outcome and VA, AVB, CK-MB increases, and histopathological lesions were secondary outcomes. Based on previous studies [8,11,12], we estimated 75% LET for SS + CIR animals. The study was designed to detect interventions with considerable effect on LET, capable of rescuing at least 2/3 of lethal arrhythmias (i.e., reducing LET at least to 25%). Sample sizes of *n* = 14 were calculated for these anticipated incidences, α = 0.05 and power 80%, using a sample size calculator (available at https://clincalc.com/stats/samplesize.aspx, accessed on 2 August 2025). A smaller sample size (*n* = 10) was used for the SHAM group to reduce animal use, as this group invariably shows 0% VA, AVB, and LET [8,11,12].

### 4.8. Statistical Analysis

Data on VA, AVB, and LET occurrences were represented as percentages and statistically analyzed using Fisher’s exact test using the Prism 8.0 program (GraphPad Software Inc., La Jolla, CA, USA). The measurements of concentration for CK-MB were made, and the results were reported as mean ± standard error of mean (SEM). Using Prism for statistical analysis, the data were subjected to an Analysis of Variance Test. When *p* < 0.05, the results were deemed statistically significant.

## 5. Conclusions

The present study’s results indicate that EPI treatment, at a dose of 15 mg/kg, reduced incidence of AVB and LET, oxidative stress, and myocardial injuries in rats that were subjected to the CIR protocol. As a result, it seems that EPI treatment is a viable and promising pharmacotherapeutic approach to lower the incidence of serious and fatal arrhythmias caused by ischemic heart diseases, such as AMI in humans.

## Figures and Tables

**Table 1:** Molecular affinity parameters of EPI with 5ZK8, 4U14, 6DT0, 7KL5, and 8E59 targets.

Complex (Ligand-Protein)	ΔG_bind_^a^ (kcal.mol^−1^)	Number of Runs	Standard Deviation	Ligands Interactions with Residues of Proteins ^b^	Function of the Macromolecule
EPI-4U14	−8.6	100	0.438	Ser A: 151, Tyr A: 529, Asp A: 147, Ile A: 116, Tyr A: 148, Tyr A: 533, Cys A: 532, Asn A: 152, Trp A: 503, Phe A: 239, Tyr A: 506, Val A: 155, Ala A: 238, Leu A: 225, Thr A: 231, Ala A: 235, Thr A: 234, Trp A: 199, Asn A: 507	Human GPCR involved in smooth muscle contraction, glandular secretions, vomiting induction, and cardiovascular regulation, as its activation in endothelial cells stimulates nitric oxide production, promoting vasodilation and blood pressure control.
EPI-5ZK8	−8.4	100	0.443	Ser A: 107, Thr A: 190, Tyr A: 104, Phe A: 195, Ala A: 191, Trp A: 400, Val A: 111, Ala A: 194, Asn A: 108, Trp A: 155, Thr A: 187, Val A: 407, Asn A: 404, Tyr A: 403, Tyr A: 426, Asp A: 103 and Tyr A: 430	Human muscarinic acetylcholine receptor M_2_, a G protein-coupled receptor (GPCR) that plays a crucial role in regulating heart rate by slowing conduction in the sinoatrial and atrioventricular nodes.
EPI-6DT0	−7.6	100	0.219	Thr D: 361, Tyr D: 362, Glu D: 358, Glu C: 358, Ca A: 501, Glu B: 358, Glu A: 358, Thr A: 361, Tyr B: 362, Trp A: 354, Tyr C: 362, Trp C: 354, Tyr A: 362	A transmembrane protein critical for cellular homeostasis, 6DT0 acts as the main channel allowing selective entry of calcium ions into the mitochondrial matrix, regulating essential processes such as ATP production, intracellular signaling, and activation of apoptotic pathways. In cardiac cells, it controls the energy production necessary for heart contraction, maintaining cardiac rhythm and function, and influencing apoptosis sensitivity under stress conditions, such as ischemia–reperfusion.
EPI-8E59	−6.8	100	0.161	MET A: 1091, GLN A: 1026, TYR A: 1448, PHE A: 1095, THR A: 1099, SER A: 1098, MET A: 1449, MET A: 1144, THR A: 1022, THR A: 1023, ILE A: 1445	A membrane receptor is essential for regulating calcium influx in excitable cells, especially cardiomyocytes and neurons, where it controls muscle contraction, electrical conduction, and neurotransmitter release.
EPI-7KL5	−5.6	100	0.197	Lys A: 14, Phe A: 66, Gly A: 24, Ser A: 18, Ala A: 11, Glu A: 15, Gly A: 26, Phe A: 17, Asp A: 21	It regulates the activity of the cardiac ryanodine receptor RyR2, modulating calcium release from the sarcoplasmic reticulum during the excitation–contraction cycle in cardiomyocytes. This regulation is crucial for maintaining the frequency and strength of heart contractions, ensuring the synchronization of the heartbeat and efficient blood pumping. Additionally, the interaction with RyR2 is modulated by additional calcium ions, which stabilize the complex and allow fine-tuning of intracellular calcium signaling, contributing to cardiac homeostasis and preventing arrhythmias.

^a^ Power bond in best conformation. ^b^ Obtained with BIOVIA Discovery Studio Visualizer.

## Data Availability

The data will be available upon justified request and agreement of the authors.
